# The aldehyde group of gossypol induces mitochondrial apoptosis via ROS-SIRT1-p53-PUMA pathway in male germline stem cell

**DOI:** 10.18632/oncotarget.22044

**Published:** 2017-10-24

**Authors:** Xin He, Chongyang Wu, Yanhua Cui, Haijing Zhu, Zhiming Gao, Bo Li, Jinlian Hua, Baoyu Zhao

**Affiliations:** ^1^ College of Veterinary Medicine, Northwest A&F University, Yangling 712100, Shaanxi, China; ^2^ College of Veterinary Medicine, Shaanxi Centre of Stem Cells Engineering & Technology, Northwest A&F University, Yangling 712100, Shaanxi, China

**Keywords:** gossypol, male germline stem cell (mGSCs), apoptosis, mitochondrion, reactive oxygen species (ROS)

## Abstract

As a widely grown economic crop, cotton is the major oil and protein resource for human and livestock. But the highly toxic of gossypol in cottonseed severely restricts its effective utilization, consequently creating huge resource waste. Previous studies have shown the male germline stem cells were the most vulnerable cells in gossypol damages, but the mechanism was still unclear. We found gossypol induced cell viability decline resulted from apoptosis. And the increase of Caspase-9 activity in gossypol treatment hinted the mitochondrial apoptosis. So the mitochondrial dysfunction was confirmed by the decreased mitochondrial membrane potential and ATP concentration. We found the higher intracellular H_2_O_2_ level did not accompany with the O_2_^·-^ associated increase in gossypol-treated, which indicated that gossypol obstructed the intracellular reactive oxygen species (ROS) elimination. Manipulated gossypol-induced H_2_O_2_ level by H_2_O_2_ and α-lipoic acid, we demonstrated that the mitochondrial dysfunction resulted from the excessive intracellular H_2_O_2_. Treated with Apogossypolone (ApoG2), an aldehyde group removed derivative of gossypol, the GSH/GSSG ratio and H_2_O_2_ did not decrease. ApoG2 also did not cause the mitochondrial apoptosis. So the aldehyde group is key factor in gossypol cytotoxicity. We respectively detected the NAD^+^/NADH ratio, SIRT1 activity, the relative protein level and apoptosis. Comparing with the specific inhibitors groups, the data illustrated that gossypol induced apoptosis through SIRT1-P53-PUMA pathway. This study helped to overcome barriers of gossypol cytotoxicity, which is crucial in feed and food use of cottonseed. This also provides a reference for the gossypol derivatives using in male contraception and anticancer.

## INTRODUCTION

As one of the world’s major economic crops, global cotton acreage is basically stable at 34 million hectares, corresponding a huge cotton by-product output in recent years [1]. For example, China’s cotton byproduct output was 3127 tons including 31% cottonseed in 2014 [2]. Being a high quality and low price feed for animal husbandry, cottonseed could meet the protein sources of numerous animals and human. However the highly toxic of (±)-gossypol (gossypol) in cottonseed severely restricts its effective use as food and fodder, consequently creating a huge waste of resources [3–5]. However, gossypol and its derivatives also have potential therapeutic use such as antineoplastic and male contraceptive [6].

As a natural product isolated from the cotton (*Gossypium*) seeds [7], gossypol is a yellow phenolic aldehyde that promotes several toxic effects in vertebrates but provides the cotton plant with resistance to pests [8]. The cottonseed may contain concentrations greater than 14,000 mg/kg total gossypol and 7,000 mg/kg free gossypol [9, 10].

Several studies showed that free gossypol inhibits spermatogenesis in different species of animals such as rat, mouse, goat and human [11]. Thirty male lambs of 3-4 months of age were fed with 40% cottonseed meal for 180 days; gossypol accumulates produced testicular weight reduced, vacuolation of seminiferous tubules, seminiferous epithelium damaged, spermatogonial cells absented, and sperm disappeared. Gossypol induces cell death via apoptosis [12–14], autophagy [15–17] and endoplasmic reticulum stress [18] in various types of cancers such as pancreatic and bladder cancer cells [19]. The signaling studies revealed that gossypol treatment upregulated microRNA miR-15a [20], suppressed cyclin-A2/Akt/FOXO3a signaling [21] or suppressed the mTOR signaling [22].

We have to confront the male reproductive damage of gossypol for its food and fodder toxicity. A number of methods have been developed to remove gossypol from cottonseed including ferrous sulfate treatment, calcium hydroxide treatment, microbe fermentation and so on, but these methods have the high cost and low efficiency disadvantages [8, 23, 24]. Therefore, we converted the mindset, focusing on the studies of its toxicological mechanism, found an applied technology to relieve and utilize the toxic effect of gossypol finally. Thus it is indeed necessary to systematically study the male reproductive toxicology of gossypol, finding the key interlocking proteins to alleviate its toxicity side-effect for the feed usage of cottonseed meal and the anti-tumor clinic therapy of gossypol derivatives.

The toxicity of gossypol aldehyde group have not been specifically studied, however, the previous reports showed that aldehyde group could promote a reactive oxygen species (ROS) eliminate obstacle via exhausting the intracellular glutathione (GSH) [25, 26]. The majority of intracellular ROS are the mitochondrial respiratory chain leaked O_2_^·-^ that should be disproportionated to H_2_O_2_ by Superoxide Dismutase (SOD), and finally eliminated by the GSH dominated redox systems [27, 28]. So we respectively chose Dihydroethidium (DHE) and DCF-DA (DCF) as the probes of O_2_^·-^ and H_2_O_2_. The higher DCF significantly increase synchronizes with a DHE no significant difference phenomenon, which reflects the ROS accumulation and eliminate obstacle.

We specialized in spermatogonia toxicity of gossypol. But the goat male germline stem cells (mGSCs) have only accounts for 0.3% in testicular cells, and there is not widely accepted marker to sort the mGSCs by flow cytometry or magnetic cell sorting. So we applied an immortalized mGSCs line called mGSCs-I-SB that has established in our laboratory [29]. First, we demonstrated that gossypol induced mGSC apoptosis in phenomenon, and further explored the mechanism. Secondly, based on the screening of cell pathways we explored, some high efficiency and low toxicity micromolecule drugs will be find to alleviate the cytotoxicity of gossypol. It not only alleviated the male reproductive toxicity of gossypol using as animal feed, but also might provide a preliminary reference for gossypol and its ramifications to ameliorate the spermatogenic damages in anticancer therapy.

## RESULTS

### Gossypol inhibited cell viability in a dose- and time-dependent manner

**Figure 1:**
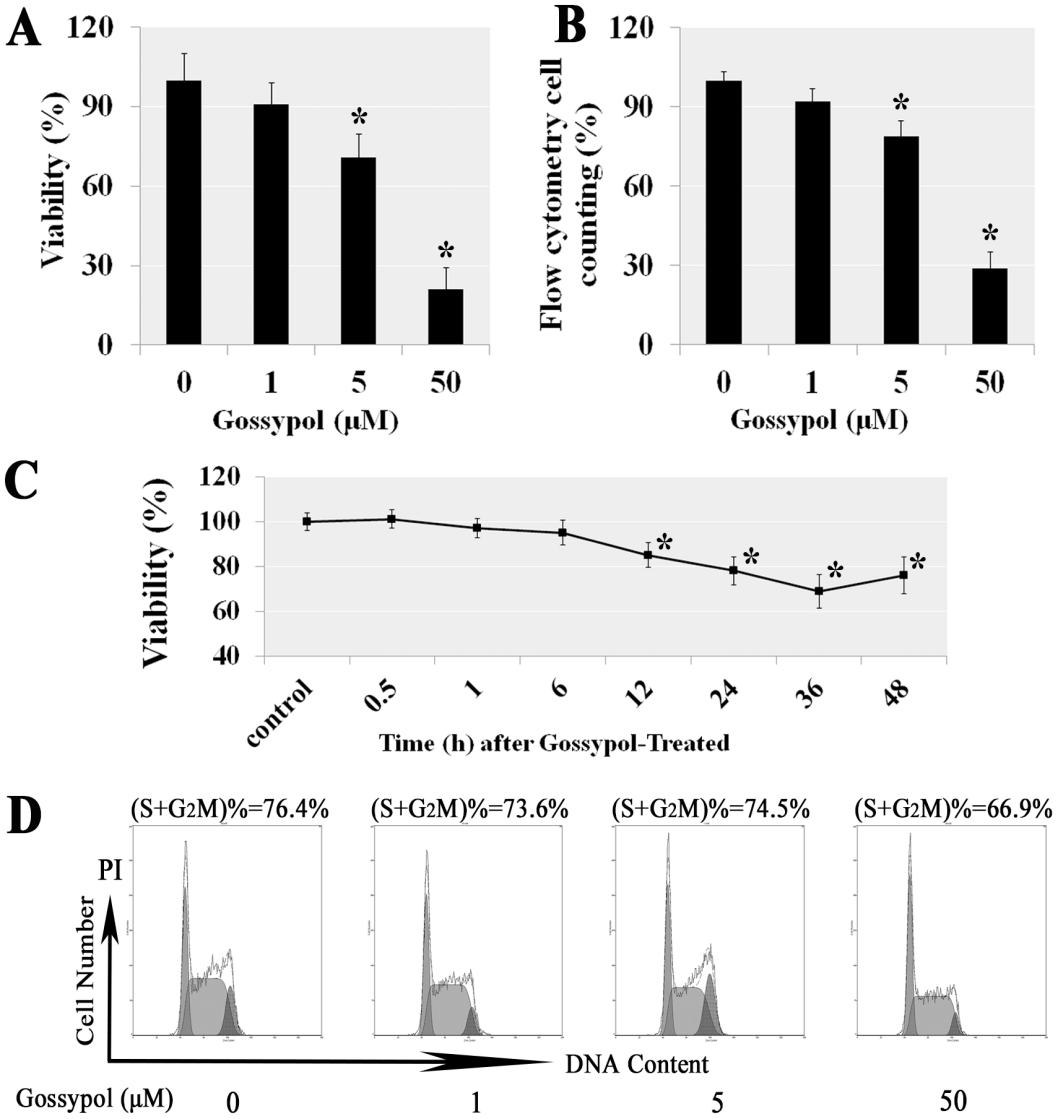
The viability of mGSCs was significantly inhibited in a dose- and time-dependent manner by gossypol The mGSCs were respectively treated with 0, 1, 5 and 50 μM concentrations gossypol for 36 h. Cell viabilities were determined using CCK-8 assay **(A)**, and Flow cytometry **(B)**. We confirmed the cell viabilities were significantly decreased in 12 h and later after 5 μM gossypol treatment in using WTS-8 assay **(C)**. However, the DNA content analysis showed that gossypol did not affect the cell cycle, it meant that the viability decrease did not result from the cell proliferation **(D)**. Thus gossypol suppressed the cell viability in a dose- and time-dependent way in mGSCs.

In order to confirm the dosage toxicity effect of gossypol, the mGSCs were respectively treated with 0 μM, 1 μM, 5 μM and 50 μM gossypol for 36 h. Cell viabilities were measured by Cell Counting Kit-8 (CCK-8) and Flow cytometry cell counting. We found the cell viabilities and numbers decrease was proportional to the increased of gossypol concentration, and the results also showed that it started occurrence the statistical difference in 5 μM (Figure 1A and 1B). We also confirmed the cell viabilities were down in the dumps at 36 h after 5 μM gossypol treatment by CCK-8 assay (Figure 1C). However, the DNA content cell cycle analysis demonstrated that almost same percentages of cells at S and G2M phases, which illustrated that same percentage cells entered in the division stage with or without gossypol treatment, so the gossypol did not inhibit the cell cycle (Figure 1D). It meant that the viability decrease did not result from the cell proliferation. Thus these findings indicated that mGSC viability was inhibited by gossypol in a dose- and time-dependent manner.

### Gossypol caused mGSC apoptosis

**Figure 2:**
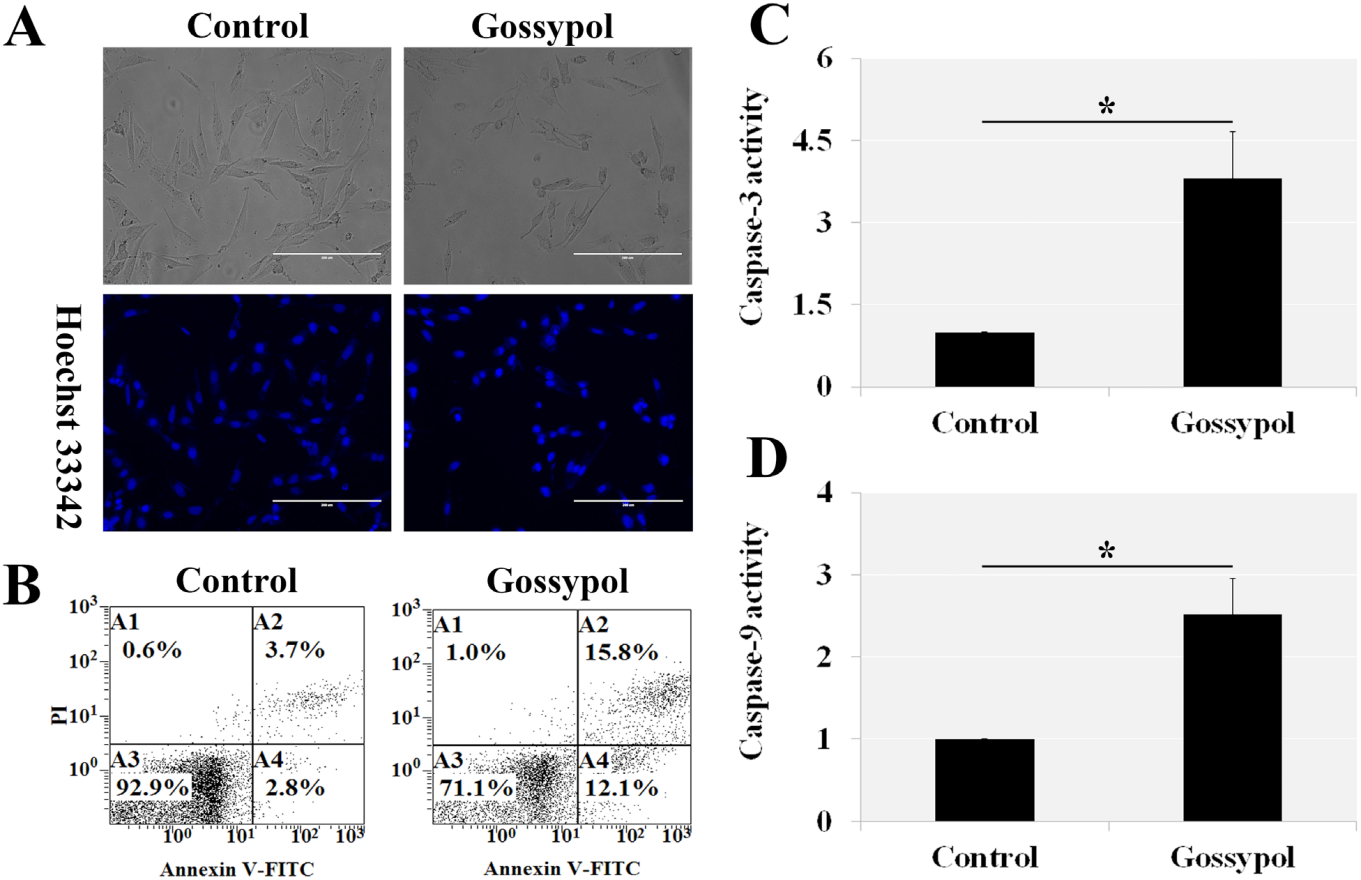
Gossypol induced mGSC apoptosis After 5 μM and 36 h gossypol treatment, the mGSCs were stained by Hoechst 33342 **(A)** or quantitative analyzed by Annexin V-FITC/PI Analysis **(B)**. Gossypol treatment increased the Caspase-3 and Caspase-9 activity, which confirmed the cell apoptosis results (**C** and **D**). The findings indicated the gossypol induced mGSC apoptosis, and increased Caspase-9 activity hinted the apoptosis from the mitochondrial apoptotic signaling pathways.

The Hoechst 33342 staining (Figure 2A) and Annexin V-FITC analysis (Figure 2B) indicated that 5 μM gossypol induced more than 20% cells apoptosis. Gossypol treatment increased the Caspase-3 and Caspase-9 activity, which confirmed the cell apoptosis results (Figure 2C and 2D). Thus gossypol suppressed the viability might because of the gossypol-induced apoptosis. The rising of Caspase-9 activity manifested the gossypol-induced apoptosis was from the mitochondrial apoptotic signaling pathways.

### Gossypol induced the mitochondrial dysfunction

**Figure 3:**
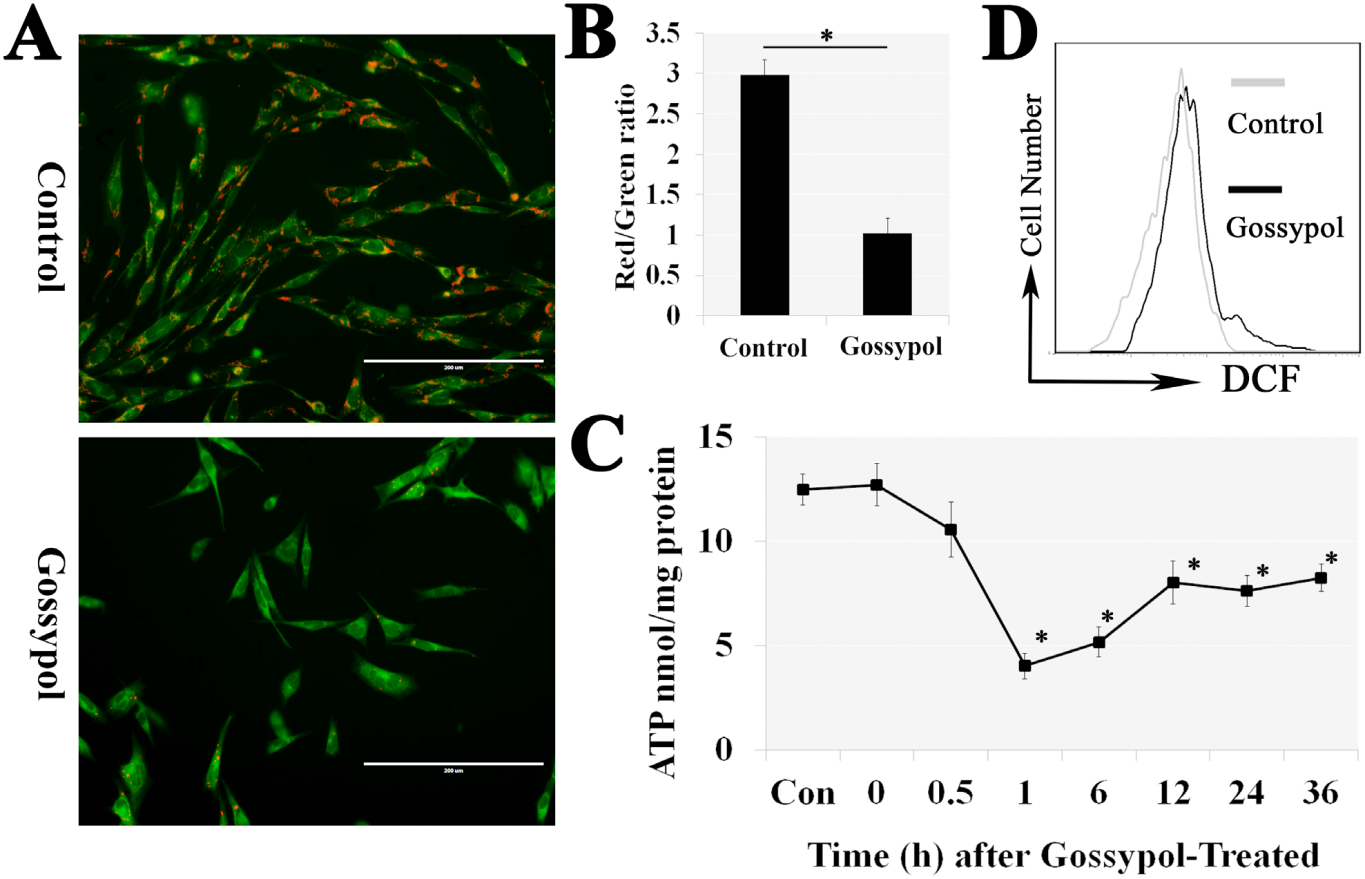
Gossypol caused mitochondrial dysfunction and the excessive intracellular ROS Cells were exposed to 5 μM gossypol for 6 h, and stained with JC-1 to measure the ΔΨm (**A** and **B**). We also measured the intracellular ATP concentration. The results exhibited bottomed out at 1 h after gossypol treatment and partly began to rise again **(C)**. The ΔΨm reduction and ATP concentration decrease manifested a gossypol-induced mitochondrial dysfunction, which may cause by the excessive intracellular H_2_O_2_ **(D)**.

Since the Caspase-9 was active by gossypol (Figure 2D), we assumed that the apoptosis was caused by the mitochondria dysfunction. Thus, the mGSCs were exposed to 5 μM gossypol for 6 h, and then measure the mitochondrial membrane potential (ΔΨm) by JC-1. The punctate red fluorescence represents the potential-dependent aggregate form of JC-1 in the mitochondria of healthy cells that have higher ΔΨm, diffuse green fluorescence represents the monomeric form of JC-1 in the cytosol of unhealthy cells that have depolarized mitochondria (lower ΔΨm). So the Red/Green ratio was a marker of ΔΨm. Gossypol treatment significantly reduced the ΔΨm (Figure 3A and 3B). We also measured the intracellular adenosine triphosphate (ATP) concentration, which bottomed out at 1 h after gossypol treatment and automatic recovered to almost 50% compared with the control group (Figure 3C). The findings manifested a gossypol-induced mitochondrial dysfunction, which may cause by the excessive intracellular ROS detected by DCF (Figure 3D). The findings indicated gossypol caused a mitochondrial dysfunction.

### Gossypol obstructed ROS elimination

**Figure 4:**
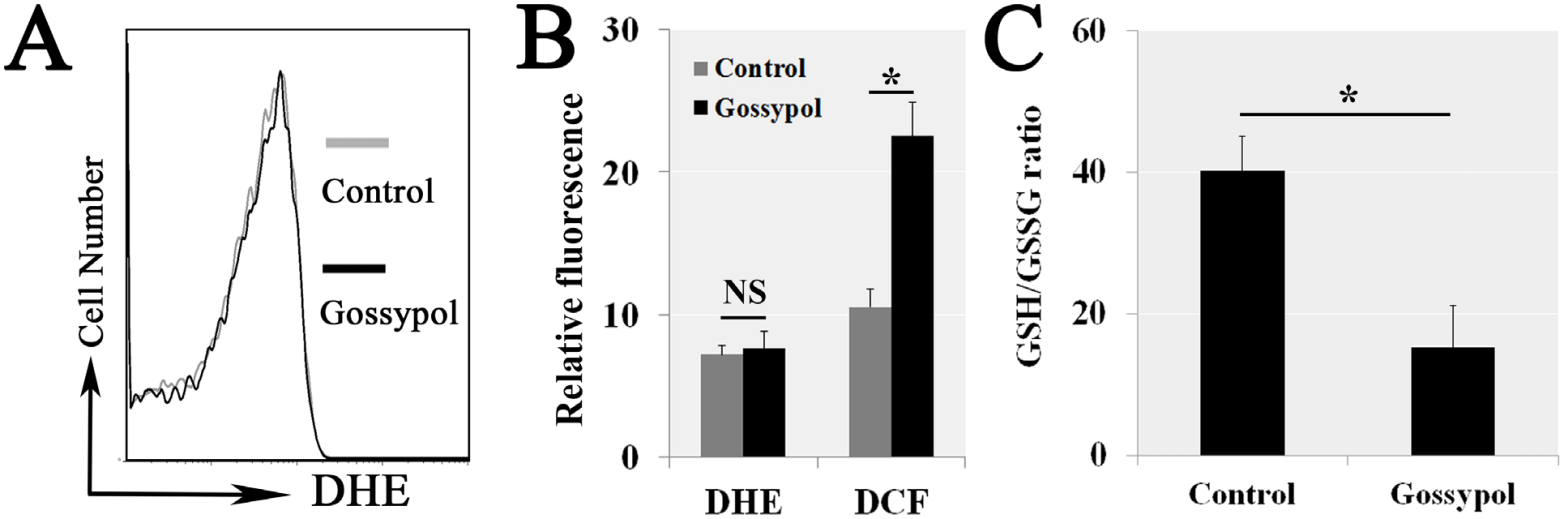
Gossypol obstructed the intracellular ROS elimination Cells were exposed to 5 μM gossypol for 6 h, then stained with Dihydroethidium (DHE) to measure O_2_^·-^ by flow cytometry **(A)**. Then we quantitative analyzed the intracellular H_2_O_2_ concentration by DCF. The results of DHE (O_2_^·-^) did not illustrate significant differences, but DCF (H_2_O_2_) increased in gossypol treatment **(B)**. The H_2_O_2_ produced from O_2_^·-^ by the SOD, so gossypol obstructed the intracellular ROS elimination. The GSH/GSSG ratio, the marker of ROS elimination, significantly decreased in gossypol-treated cells **(C)**.

Since the apoptosis and mitochondrial dysfunction were associated with an excessive intracellular ROS, we further explored the source of these ROS and whether it induced the apoptosis. Cells were exposed to 5 μM gossypol for 6 h, then stained with DHE to measure O_2_^·-^ by flow cytometry (Figure 4A). Then we quantitative analyzed the intracellular ROS (measured by DCF) and O_2_^·-^ (measured by DHE); the results of DHE did not illustrate significant differences, contrasted with DCF that increased in gossypol treatment (Figure 4B). The most O_2_^·-^ were produced by mitochondria, thus no significant differences of DHE manifested gossypol treatment did not induce the excessive ROS generation. However, the most H_2_O_2_ produced from O_2_^·-^ by the SOD, so only H_2_O_2_ increase demonstrated gossypol obstructed the intracellular ROS elimination. Therefore, we considered there was a certain relationship between intracellular ROS and GSH/GSSG ratio. The following experiments proved this characteristic, GSH/GSSG ratio significantly decreased in gossypol-treated cells compared with the control (Figure 4C). Gossypol-induced excessive ROS resulted from the ROS elimination obstructed.

### Gossypol-induced ROS resulted in a mitochondrial dysfunction

**Figure 5:**
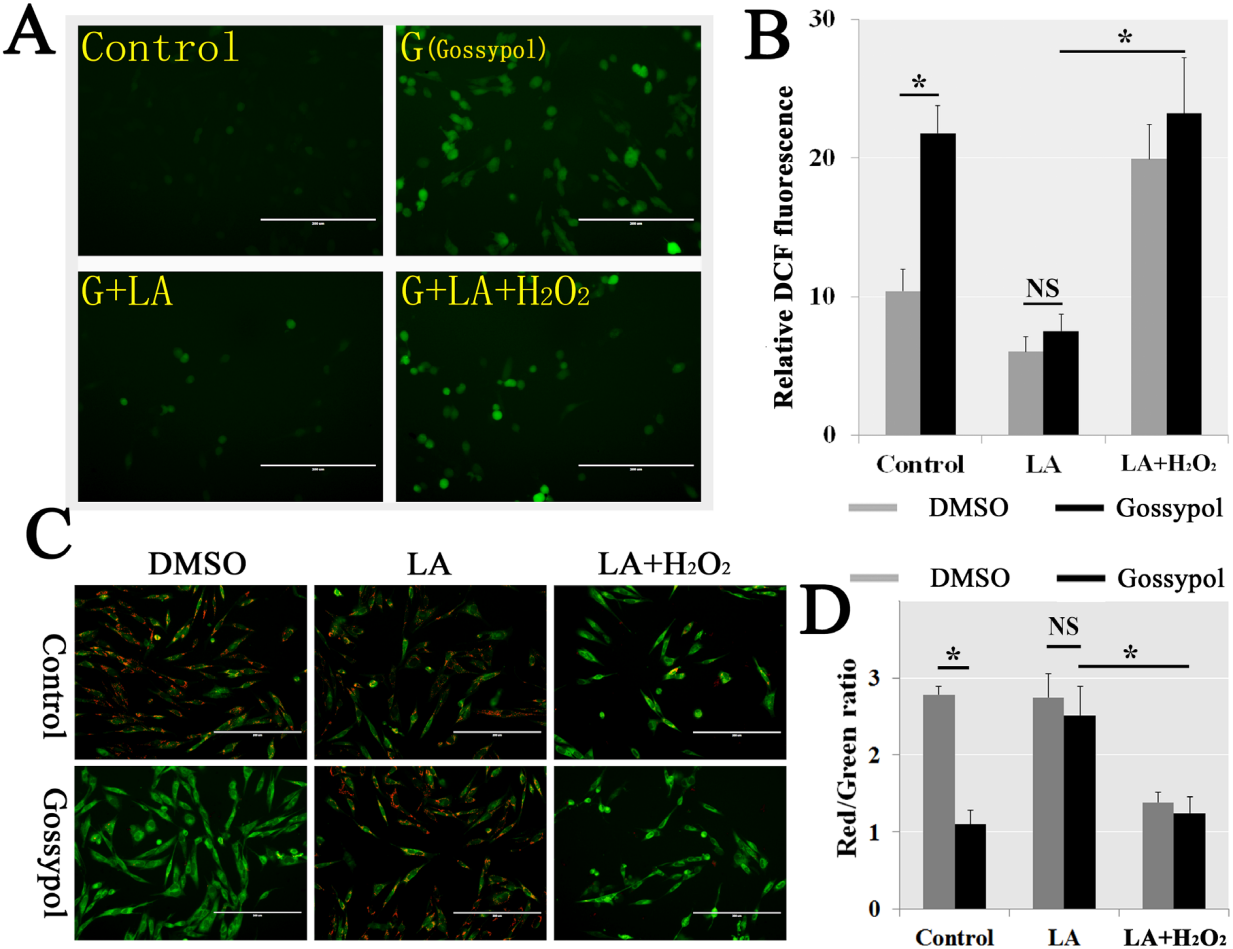
Gossypol-induced ROS caused mitochondrial dysfunction We pretreated with 2 mM LA (α-lipoic acid, a ROS scavenger) or LA and H_2_O_2_ (a ROS inducer) for 1 h to eliminate the ROS, and then added 5 μM gossypol at 6 h after pretreated in mGSCs. The samples were measured by DCFH-DA to confirm the ROS scavenged (**A** and **B**). The ΔΨm was determined by JC-1. The Red/Green ratio had a positive significant associated with the ROS level, and there did not exhibited a mitochondrial dysfunction when we eliminated the gossypol-induced ROS by LA (**C** and **D**). We found that the requirement for mitochondrial dysfunction was gossypol-induced ROS.

We have added 2 mM LA (α-lipoic acid, a ROS scavenger) and/or 100 μM H_2_O_2_ (a ROS inducer) 1 h before gossypol treatment to downregulate and/or upregulate the ROS. The DCF prober demonstrated that intracellular ROS was significantly decreased in LA pretreatment and increased in H_2_O_2_ and LA group (Figure 5A and 5B). The JC-1 stain illustrated the Red/Green ratio had a positive significant association with the ROS level, and there was not exhibited a mitochondrial dysfunction when we eliminated the gossypol-induced ROS by LA (Figure 5C and 5D). Therefore, we confirmed that the gossypol-induced mitochondrial dysfunction was the consequences of intracellular ROS elimination obstructed.

### Aldehyde group of gossypol was the key factors in GSH depleted and related ROS eliminated obstruction

**Figure 6:**
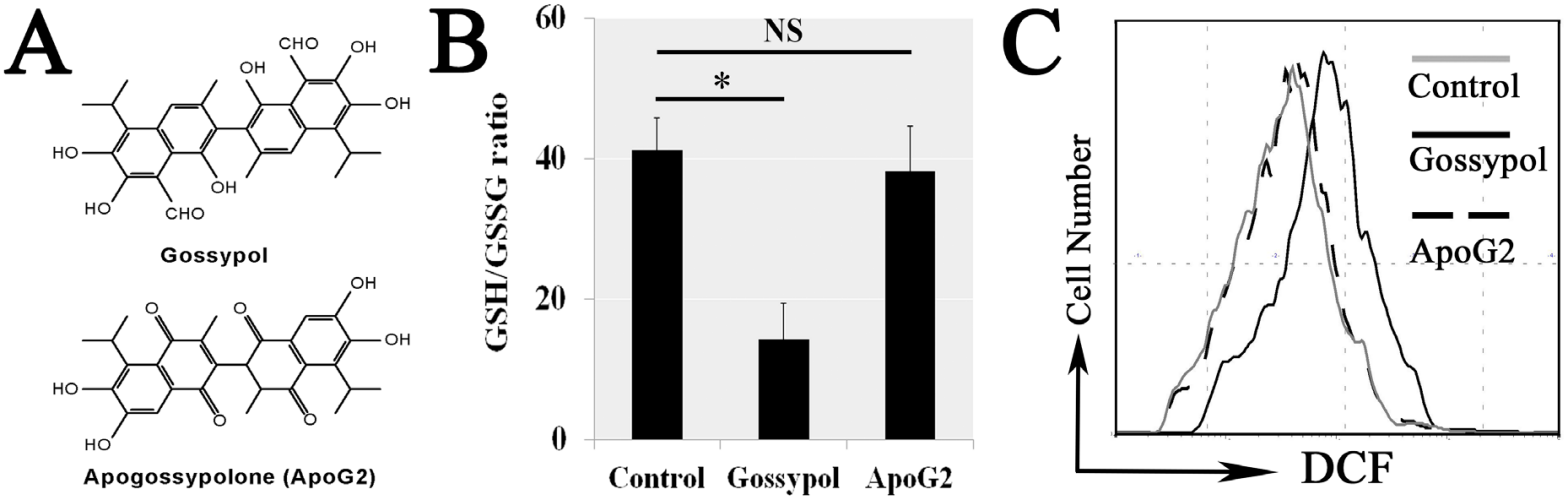
The aldehyde group of Gossypol induced GSH depleted and ROS eliminated obstruction Apogossypolone (ApoG2) is a derivative that removed the aldehyde group of gossypol in structural formulas **(A)** (ChemWindow V6.0, Bio-Rad Laboratories). The mGSCs were separately treated with 5 μM gossypol, ApoG2 or DMSO for 6 h. The GSH/GSSG ratio did not decrease in ApoG2 treatment contrasting with the gossypol **(B)**. We also found ApoG2 did not obstruct intracellular ROS elimination **(C)**.

Apogossypolone (ApoG2) is a derivative of gossypol that removed the aldehyde group in structural formulas (Figure 6A) (ChemWindow V6.0, Bio-Rad Laboratories). The mGSCs were separately treated with 5 μM gossypol, 5 μM ApoG2 or 5 μM Dimethyl sulfoxide (DMSO) for 6 h. The GSH/GSSG ratio did not decrease in ApoG2 treatment contrasting with the gossypol (Figure 6B). We also found ApoG2 did not obstruct intracellular ROS elimination (Figure 6C). Therefore, it is aldehyde group that triggered the GSH depleted and related ROS eliminated obstruction in gossypol treatment.

### The requirement for gossypol-induced mitochondrial apoptosis was its aldehyde group

**Figure 7:**
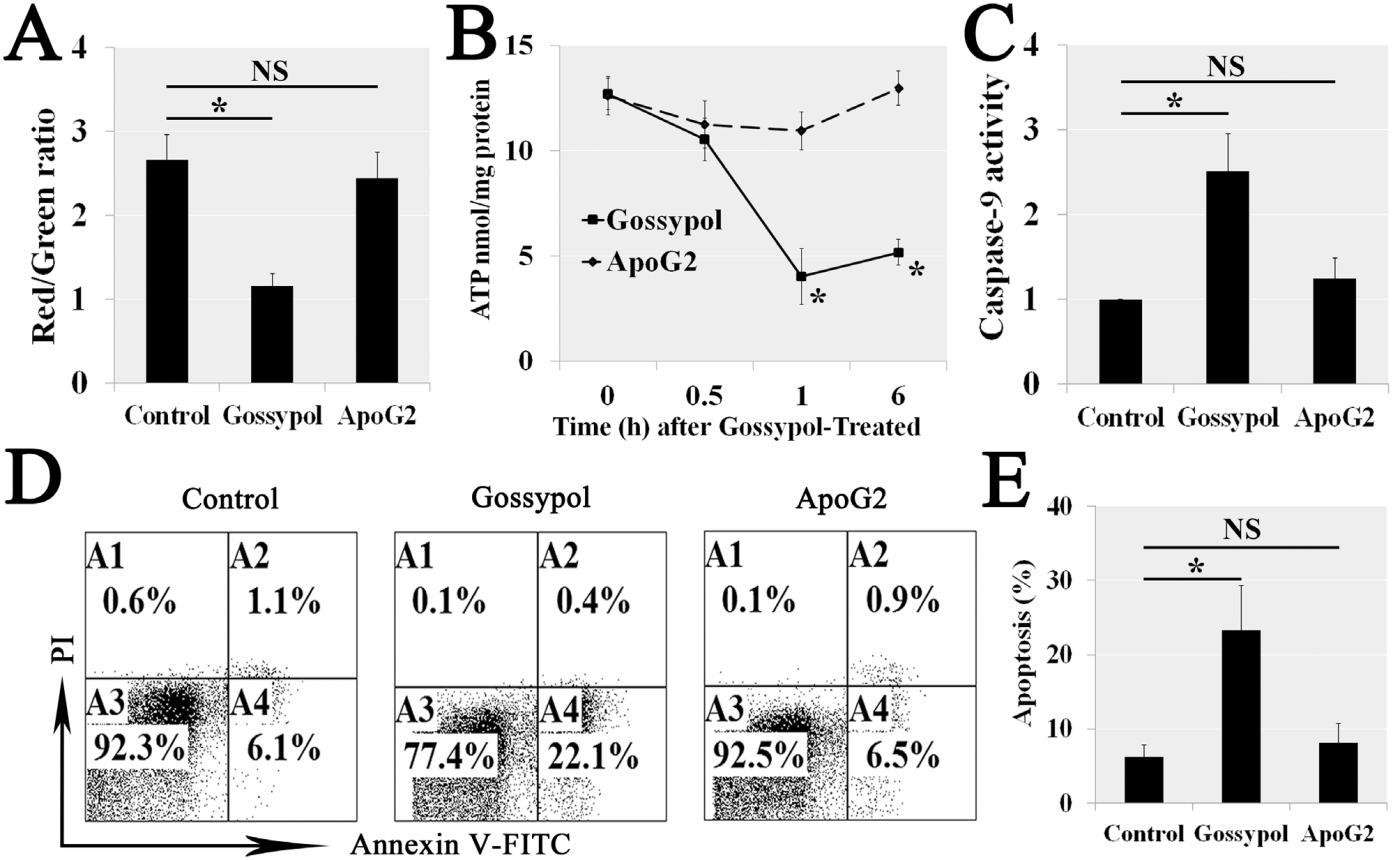
The aldehyde group of Gossypol induced mitochondrial dysfunction and related apoptosis We confirmed that ApoG2 treatment did not induce the mGSCs ΔΨm **(A)** and ATP **(B)** decrease. The cytoplasm protein concentration of cleaved Caspase-9 (c-Cas9) increased in gossypol treatment but ApoG2 did not affect those proteins in mitochondria apoptosis pathway **(C)**. We also measured the cell apoptosis by Annexin V-FITC/PI Analysis, the results showed ApoG2 did not induce apoptosis compared with gossypol (**D** and **E**).

Since aldehyde group cased a ROS eliminated obstruction, we further ruled out the ApoG2 mitochondrial dysfunction, because ApoG2 treatment did not induce the mGSCs ΔΨm (Figure 7A) and ATP (Figure 7B) decrease. The cytoplasm protein concentration of cleaved Caspase-9 (c-Cas9) increased in gossypol treatment, but ApoG2 did not affect those proteins in mitochondria apoptosis pathway (Figure 7C). We also measured the apoptosis by Annexin V-FITC/PI Analysis, the results showed ApoG2 did not induce apoptosis compared with gossypol (Figure 7D and 7E). Thus we confirmed that other parts of gossypol removed the aldehyde group did not cause the mitochondrial apoptotic signaling pathways. Therefore, it is the aldehyde group of gossypol that causes its cytotoxicity.

### Gossypol induced apoptosis via SIRT1-P53-PUMA pathway

**Figure 8:**
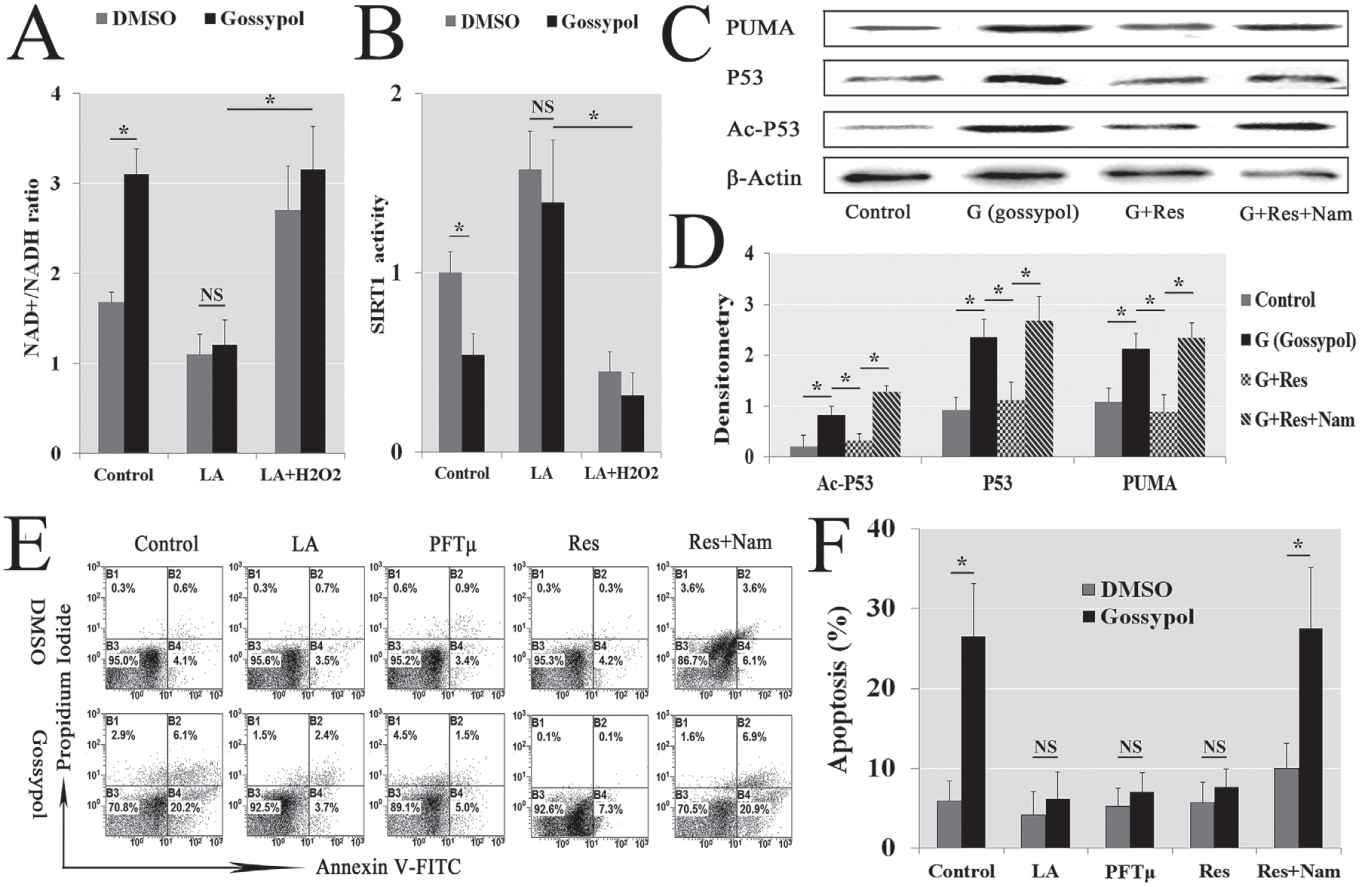
Gossypol induced apoptosis through SIRT1-P53-PUMA pathway The mGSCs were pretreated with LA and/or H_2_O_2_ for 1 h, and then added 5 μM gossypol. The NAD^+^/NADH ratio decreased in gossypol treatment, recovered in LA pretreatment contrasting with the gossypol, but H_2_O_2_ pretreated could abolish the Res effect **(A)**. SIRT1 enzyme activity was inhibited in gossypol treatment, and increased in LA pretreating and abolished in LA + H_2_O_2_ coprocessing **(B)**. To illuminate the pathway of gossypol cytotoxicity, mGSCs were pretreated with Res (Resveratrol, a SIRT1 activator) and/or Nam (Nicotinamide, a SIRT1 inhibitor) for 1 h, and then added 5 μM gossypol, after treating with gossypol 6 h Western blotting was used to demonstrate the protein concentration of Ac-P53, P53 and PUMA, the relatively quantified by ImageJ (V1.48d) indicated intracellular ROS concentration could affect this pathway (**C** and **D**). The Annexin V-FITC/PI analysis demonstrated gossypol induced 27% cell apoptosis, but LA, PFTμ (Pifithrin-μ, a p53 inhibitor) and Res pretreatment almost completely alleviated the gossypol-induced apoptosis. Pretreating with Res and 100 nM Nam abolished the Res alleviated apoptosis (**E** and **F**), which confirmed the above results.

**Figure 9:**
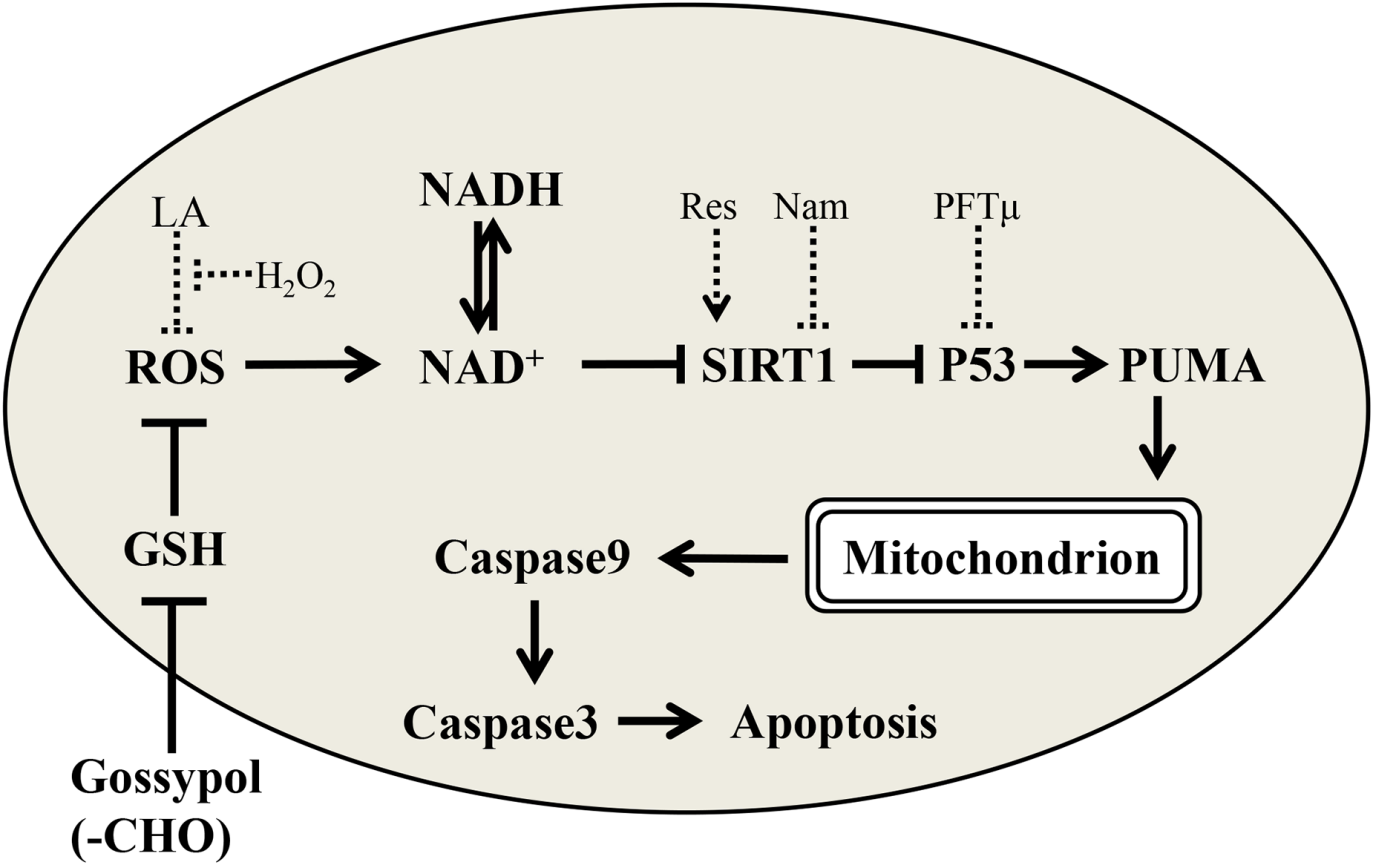
Schema showed the possible mechanism that the aldehyde group of gossypol induces apoptosis starting from the GSH depleted and following ROS-induced mitochondria dysfunction via SIRT1-p53-PUMA pathway

To detail illuminate the contact of gossypol-induced ROS and mitochondrial apoptosis, we detected the intracellular ROS sensitive NAD^+^/NADH ratio (NAD: Nicotinamide adenine dinucleotide). The data showed that the ratio was increased in gossypol-treated, but decreased in LA pretreatment contrasting with only gossypol treatment, so the Nam (Nicotinamide, a SIRT1 inhibitor) coprocessing could abolish the LA effect (Figure 8A). The SIRT1 (Silent mating type information regulation 2 homolog-1) enzyme activity was inhibited in gossypol treatment, and no significant difference increased in LA pretreated, but H_2_O_2_ could abolished the LA effect (Figure 8B). To illuminate the pathway of the cytotoxicity, we further used Western blotting to demonstrate the protein concentration of Ac-P53 (acetylization-P53), P53 and PUMA (P53 upregulated modulator of apoptosis). The relatively quantified by ImageJ (V1.48d) indicated gossypol-treated could affect the intracellular concentration of these protein by SIRT1-P53-PUMA pathway (Figure 8C and 8D). At last, the inhibitor-driven reversing verification method has been used in apoptosis analyses. The Annexin V-FITC/PI Analysis demonstrated gossypol induced 27% cell apoptosis, but LA, PFTμ (Pifithrin-μ, a P53 inhibitor) and Res (Resveratrol, a SIRT1 activator) pretreatment almost completely alleviated the gossypol-induced apoptosis; but pretreating with Res and 100 nM Nam abolished the Res alleviated apoptosis (Figure 8E and 8F). Thus we illustrated that gossypol-induced ROS activated the SIRT1-P53-PUMA pathway, which induced the mitochondrial apoptosis (Figure 9).

## DISCUSSION

Large mass cottonseeds are being harvested as a by-product of cotton production in many areas of the world. But it is a thorny issue that cottonseeds contained gossypol, which have physiology safety problems such as high cytotoxicity that observers noted apoptosis induced in the cells with fast-cycle. Thus gossypol also has been developed for anticarcinogen, male contraceptive and female hormone-dependent cell proliferation diseases [30]. Because of its anti-tumor application prospects, the current studies of cytotoxic mechanism of gossypol and its derivatives based on tumor cells [31]. Few studies have addressed the specific molecular mechanisms of gossypol damage to spermatogonia, however, most studies on reproductive toxicity of gossypol still major concerned in its toxic phenomenon [32].

In this study, we focused on analyzing the mechanism of gossypol reproductive toxicity. Using an aldehyde-removed derivative as control verification, we found that the aldehyde group was the key of gossypol-induced ROS eliminate obstacles and GSH/GSSG ratio decline. Previous studies have reported that aldehyde compounds, such as formaldehyde and acrolein, could apt to addition reaction with intracellular GSH resulted in multitudinous adducts [33, 34]. Our data showed that intracellular ROS came from an eliminate obstacles, then we verified the GSH/GSSG ratio decrease. We speculated the gossypol unceasingly consumes the intracellular GSH via addition reactions, interrupted the GSH/GSSG circulation for ROS elimination. But the aldehyde-removed derivative did not cause mitochondrial apoptosis. Thus we confirmed the cytotoxicity of gossypol rooted in the intramolecularly aldehyde, which consumed intracellular GSH caused a ROS eliminate obstacles.

We further illustrated the signal transduction pathway between the gossypol-induced ROS eliminate disorder and mitochondrial apoptosis. We found that excessive ROS increased the intracellular NAD^+^/NADH ratio, which inhibited the activity of SIRT1. The SIRT1 deacetylated P53 can be readily degraded by ubiquitination [35, 36]. Therefore the inhibition of SIRT1 activity increase intracellular P53 concentration. The P53 raised PUMA expression [37, 38], which caused mitochondrial permeablity transition pore (mPTP) and related apoptosis [39]. We illuminated the above pathway on the basis of detected the expression and activated these proteins, and further applied various inhibitors to verify the conclusion.

Since intracellular ROS and followed mitochondrial dysfunction were the key mechanism of gossypol reproductive toxicology, we used Res to try regulation them. Data showed that Res could relieve the gossypol-induced apoptosis of spermatogenic cells in a low concentration such as 10 μM, which has potential applications in the mitigation of feed toxicity and chemotherapy side-effect. With the development of biotechnological approaches for Res production [40], compared with the traditional methods to remove the gossypol from cottonseed, it is economic and good effect for large-scale application in Res added directly.

In conclusion, we studied the key mechanism in gossypol male reproductive toxicology. The above data suggested that the cytotoxicity was attributed to intramolecular aldehyde groups, which consumed intracellular GSH caused a ROS eliminate obstacles; the excessive ROS caused mitochondrial apoptosis via SIRT1-P53-PUMA pathway (Figure 9). This clear mechanism could help us to solve the gossypol toxic; for example, the ROS scavenger or SIRT1 activator, could affected target protein, which made into a feed additive and chemotherapy protection medicine to relieve the gossypol toxicology in the future. It also could provide some references to avoid side effect in gossypol derivatives using for male contraception and cancer treatment.

## MATERIALS AND METHODS

### Reagents

All reagents were purchased from Sigma Chemical Co. (St. Louis, MO, USA) unless otherwise indicated. All compounds were solubilized in dimethyl sulfoxide (DMSO) except specific statements. The steroid-free medium containing DMSO (vehicle) was used as control.

### Cell treatment

The mGSCs-I-SB were established by transfection of SV40 large T antigen and Bmi1 into primary germ line stem cells [29]. The cell line were collected and cultured with DMEM/F12 (Invitrogen) supplemented with 10% fetal bovine serum (FBS, Hyclone, USA), 0.1 mM β-mercaptoethanol (Sigma) and 2 mM glutamine (Invitrogen) at 37°C, 5% CO_2_ [29].

### Determination of intracellular ROS generation

DCF: Cells were washed with phosphate-buffered saline (PBS) before loading with 50 μM DCFH-DA (Reactive Oxygen Species Assay Kit, Beyotime Institute of Biotechnology, China) in PBS for 20 min at 37°C and then washed with PBS twice. Res and gossypol was added at the indicated concentrations and incubated for designated times, prior to immediately placed into flow cytometer (Altra, Beckman Co.) with an excitation wavelength of 488 nm and emission of 530 nm [34].

DHE: The cells were washed with PBS before loading with 5 μM DHE (Dihydroethidium Kit, Beyotime) in PBS for 30 min at 37°C. The cells were then washed with PBS twice. Res and gossypol was added at the indicated concentrations and incubated for designated times, prior to immediately placed into flow cytometer with an excitation wavelength of 488 nm and emission of 610 nm [34].

DCF and DHE related fluorescence was measured using logarithmic amplification in flow cytometer. 15000 cells were analyzed for each group, and data were reported as the mean of the fluorescence intensity.

### Cell viability assay

Cell viability was determined using a CCK-8 assay (Cell Counting Kit-8, Beyotime). Briefly, cells were seeded at 5000 cells per well (96-well plate). After 12 hours common culture, the per-planning concentration of gossypol were added. In the end of each pre-planning treated time, 10 μl WST-8 (No. C0037, Beyotime) was added to each well of 96-well plate and the plates were incubated for 2 h at 37°C in 5% CO_2_ and 95% air. Optical density (OD) was measured at 450 nm with a microplate reader [41].

### DNA content and cell cycle analysis

Collect a volume of cell suspension corresponding 1 × 10^6^ cells. Pellet the cells by centrifugation. Discard the supernatant. Wash it once by PBS. Pellet the cells by centrifugation. Discard the supernatant. Add 1 mL of reagent A and 10μL reagent B (Cell cycle Kit Cat No: CCS012A Reagent A and CCS012B Reagent B; MultiSciences Biotech Co.), blend by vortexing for 5-10 seconds. Incubate for 30 minutes at room temperature. Then we analyzed using flow cytometer and WinCycle32 (Phoenix Flow Systems, Inc. 6.16.03.F32) [42].

### Annexin V- FITC apoptosis analysis

The cells were harvested, and washed with cold PBS and cold 1× Binding Buffer. Suspending cells in cold 1× Binding Buffer to a concentration of 1×10^6^ cells/ml. Adding 100 μl of cells (1×10^5^ cells) to each labeled tube, and 5 μl Annexin V-FITC to appropriate tubes. Gently vortex each tube and incubate for 10 min at room temperature. Add 5 μl of PI solution for 5 min at room temperature, protected from light. Washing cells once with PBS and resuspended in PBS, then analyzed using flow cytometer [43].

### Mitochondrial membrane potential (ΔΨm) assay

The ΔΨm was determined using the dual-emission mitochondrion-specific lipophilic, JC-1 (Gibco-Invitrogen). The punctate red fluorescence (excitation 530 nm/emission 600 nm) represents the potential-dependent aggregate form of JC-1 in the mitochondria of healthy cells (polarized mitochondria). Diffuse green fluorescence (excitation 490 nm, emission 530 nm) represents the monomeric form of JC-1 in the cytosol of unhealthy cells (depolarized mitochondria). Cells grown on the coverslip were incubated with JC-1 (10 μg/ml) at 37°C for 15 min and washed with PBS and then mounted on Leica microscope equipped with an on-stage incubator (20/20 Technologies, Pompano Beach, FL, USA) for imaging. TRITC and GFP filter sets (Semrock) were used to detect the depolarized and repolarized mitochondria, respectively. Both color channels were overlaid in FlowJo 7.61 to measure the distribution of both repolarized and depolarized mitochondria in the field [44].

### Measurement of intracellular ATP concentration

The ATP assay kit was from Beyotime (S0027) and the assay was performed according to manufacturer’s instruction. Briefly, discarding the supernatant, cells were grounded with 200 μl lysis buffer and centrifuged at 8000g for 10 min. And then 100 μl per sample was assessed with100 μl ATP detection buffer. Luminescence was determined by amulti-fluorescence microplate. While protein concentrations were determined using a BCA Protein Assay Kit (P0012S, Beyotime) [45, 46].

### GSH/GSSG ratio and NAD^+^/NADH ratio assay

The GSH and GSSG levels were measured using a GSH and GSSG Assay Kit (S0053, Beyotime), the related assay was performed according to manufacturer’s instruction. The NAD^+^/NADH ratio were measured using a NAD^+^/NADH Assay Kit (E2ND-100, BioAssay Systems), according to the manufacturer’s instruction, we used the standard monitored colorimetrically at 565 nm to manufacture a standard curve. Then we collected 10^5^ cells each sample, washed with 4°C PBS, making the reaction system to measure the OD at 565 nm calculate the data in the standard curve [47].

### Western blot analysis

Total cell extracts were prepared from mGSCs cells, and proteins were extracted in 1×SDS–PAGE sample loading buffer. Total cell proteins were resolved by SDS–PAGE, transferred to PVDF membrane, and probed with β-Actin (1:1000, Beyotime), SIRT1 (1:1000, Cell Signaling Technology), P53 (1:1000; Cell Signaling Technology), Ac-P53 (1:1000; Cell Signaling Technology), Horse-radish peroxidase conjugated anti-rabbit IgG was used as a secondary antibody (1:1000, Beyotime). The detection was performed using the BM-Chemiluminescence blotting substrate (Roche, Shanghai, China), then the maps have been analyzed by ImageJ (V1.48d) for their integrated density [42].

### SIRT1 deacetylase activity assay

SIRT1 deacetylase activity was measured with SIRT1 Fluorimetric Drug Discovery Kits (Enzo Life Sciences). Initial deacetylation rates of SIRT1 were determined at 1 unit human recombinant SIRT1 enzyme, 25 μM deacetylase substrate, and 25 μM NAD^+^ (37°C) in the absence as control or presence of 10 μl of extracted protein, measured at 460 nm. Standard curve was produced with serially diluted-deacetylation standard. Activity was normalized to protein concentration and expressed as deacetylated product (μmol)/ protein (μg) [48].

### Caspase-3 activity assay

DEVDase caspase-3 activity was determined using Caspase-3 Activity Assay Kit (C1115, Beyotime) according to the manufacturer’s instructions. The release of free pNA (p-nitroaniline) from Caspase-3 activity was monitored colorimetrically at 405 nm for 1 h using a microplate reader. Thus the Caspase-3 activity was detected by pNA concentration (nmol)/protein (mg). Then we set control group as 1 to measure the relative Caspase-3 activity of each group [49].

### Caspase-9 activity assay

Ac-LEHD-pNA (acetyl-Leu-Glu-His-Asp p-nitroanilide) Caspase-9 activity was measured using Caspase-9 Activity Assay Kit (C1158, Beyotime) according to the manufacturer’s instructions. The release of free pNA from Caspase-9 activity was monitored calorimetrically at 405 nm for 1 h using a microplate reader. Thus the Caspase-9 activity was detected by pNA concentration (nmol)/protein (mg). Then we set control group as 1 to measure the relative Caspase-9 activity of each group [50].

### Statistical analysis

The data was presented as mean ± SEM (the standard errors of the mean) from three independent experiments; three replicates were evaluated for each experiment. Statistical comparisons were assessed with analysis of Student’s test. P <0.05 was statistically considered as a statistic significant difference. All statistical analyses were performed using the SPSS software for Windows V17.0 (SPSS, Chicago, IL, USA) [34].
